# Supplementary material for: The Effects of a Preconception Lifestyle Intervention on Childhood Cardiometabolic Health—Follow-Up of a Randomized Controlled Trial
Source: Cells. 2021 Dec 24;11(1):41. doi: 10.3390/cells11010041 (PMC8750944; doi:10.3390/cells11010041)
Supplement: Supplementary file 1 [file cells-11-00041-s001.zip › cells-1448977-supplementary.pdf]

Supporting information

“The effects of a preconception lifestyle intervention on childhood cardiometabolic health – follow-up of a RCT.”

Stijn Mintjens, Mireille N.M. van Poppel, Henk Groen, Annemieke Hoek, Ben Willem Mol, Rebecca C. Painter, Reinoud J.B.J. Gemke, Tessa J. Roseboom

Corresponding author:
Stijn Mintjens, MD

Department of Pediatrics

Department of Gynecology and Obstetrics

Emma Children’s Hospital

Amsterdam Reproduction and Development research institute

Amsterdam UMC

Vrije Universiteit Amsterdam

De Boelelaan 1117 - Room 8D-14

1081 HZ Amsterdam

The Netherlands

s.mintjens@vumc.nl

Phone: +31 20 44 45238

Table S1. Cardiometabolic outcome values of boys and girls of mothers from the intervention and control group.

		**Anthropometry**				
		N	***Intervention***	*N*	*Control*	*P-value*
**BMI (Z-score) – mean (SD)**	Boys	6	0.94 (1.66)	16	0.53 (1.11)	0.51
	Girls	10	0.55 (0.82)	12	0.75 (0.98)	0.61
**Waist circumference (cm) – mean (SD)**	Boys	6	55.1 (5.3)	16	53.4 (5.3)	0.50
	Girls	11	52.5 (3.7)	13	53.4 (5.5)	0.65
**Hip circumference (cm) – mean (SD)**	Boys	6	59.2 (6.2)	16	58.1 (6.2)	0.73
	Girls	11	57.9 (3.4)	13	58.9 (8.0)	0.70
**Body-fat (%)– mean (SD)**	Boys	5	19.9 (10.1)	14	20.2 (8.2)	0.95
	Girls	11	21.0 (7.1)	12	22.3 (10.9)	0.75
		**Cardiovascular**				
		N	*Intervention*	*N*	*Control*	*P-value*
**SBP (Z-score) – mean (SD)**	Boys	5	1.00 (0.54)	16	0.45 (0.58)	0.08
	Girls	11	0.21 (0.55)	11	0.68 (0.54)	0.06
**DBP (Z-score) – mean (SD)**	Boys	5	1.21 (0.77)	16	0.92 (0.61)	0.40
	Girls	11	0.78 (0.60)	11	1.03 (0.52)	0.31
**PWV (m/sec) – mean (SD)**	Boys	3	4.69 (1.48)	12	4.70 (1.04)	0.99
	Girls	9	4.45 (0.62)	10	4.28 (1.27)	0.70
		**Metabolic**				
		N	*Intervention*	*N*	*Control*	*P-value*
**Triglycerides (mmol/L) – mean (SD)**	Boys	3	0.46 (0.13	11	0.51 (0.19)	0.74
	Girls	4	0.90 (0.81)	6	0.56 (0.13)	0.47
**Total cholesterol (mmol/L) – mean (SD)**	Boys	3	3.69 (0.56)	11	4.04 (0.62)	0.39
	Girls	4	4.69 (0.69)	6	4.12 (0.41)	0.13
**LDL cholesterol (mmol/L) – mean (SD)**	Boys	3	1.88 (0.41)	11	2.30 (0.41)	0.15
	Girls	4	2.89 (0.40)	6	2.47 (0.39)	0.14
**HDL cholesterol (mmol/L) – mean (SD)**	Boys	3	1.59 (0.19	11	1.52 (0.29)	0.68
	Girls	4	1.40 (0.19)	6	1.40 (0.17)	0.96
**Insulin (µIU/ml) – mean (SD)**	Boys	3	4.56 (1.87)	7	4.45 (3.22)	0.96
	Girls	4	6.25 (3.94)	6	3.87 (2.63)	0.31
**Glucose (mmol/L) – mean (SD)**	Boys	3	4.73 (0.12)	11	4.61 (0.35)	0.56
	Girls	4	4.68 (0.45)	6	4.20 (0.43)	0.13
**HOMA-IR – mean (SD)**	Boys	3	0.96 (0.41)	7	0.95 (0.71)	0.98
	Girls	4	1.36 (0.97)	5	0.76 (0.57)	0.29

BMI = Body mass index, SBP = Systolic blood pressure, DBP = Diastolic blood pressure, PWV = Pulse wave velocity, LDL = Low density lipoprotein, HDL = High density lipoprotein, HOMA-IR = Homeostatic model of insulin resistance.

Table S2: Cardiometabolic outcome values of children of mothers who successfully* lost weight compared to children of mothers who did not successfully lose weight, pooled data independent of the randomization group.

		**Anthropometry**			
	N	***Successful***	***N***	*Unsuccessful*	*P-value*
**BMI (Z-score) – mean (SD)**	8	0.52 (0.73)	36	0.68 (1.15)	0.72
**Waist circumference (cm) – mean (SD)**	8	53.2 (5.3)	38	53.4 (5.3)	0.93
**Hip circumference (cm) – mean (SD)**	8	58.7 (6.5)	38	58.3 (6.5)	0.88
**Body-fat (%)– mean (SD)**	8	20.8 (6.4)	34	21.0 (9.3)	0.94
		**Cardiovascular**			
	N	*Successful*	*N*	*Unsuccessful*	*P-value*
**SBP (Z-score) – mean (SD)**	8	0.24 (0.68)	35	0.57 (0.57)	0.16
**DBP (Z-score) – mean (SD)**	8	0.98 (0.70)	35	0.93 (0.58)	0.84
**PWV (m/sec) – mean (SD)**	6	4.86 (0.76)	28	4.43 (1.08)	0.36

BMI = Body mass index, SBP = Systolic blood pressure, DBP = Diastolic blood pressure, PWV = Pulse wave velocity.

* Successful was defined as: women who lost >5% of their original body weight or achieved a BMI <29 kg/m2 during the 24 months LIFE*style* study period, independent of assigned group at randomization.
